# Changes in health status, workload, and lifestyle after starting the COVID-19 pandemic: a web-based survey of Japanese men and women

**DOI:** 10.1186/s12199-021-00957-x

**Published:** 2021-03-22

**Authors:** Machi Suka, Takashi Yamauchi, Hiroyuki Yanagisawa

**Affiliations:** Department of Public Health and Environmental Medicine, The Jikei University School of Medicine, 3-25-8 Nishi-Shimbashi, Minato-ku, Tokyo, 105-8461 Japan

**Keywords:** COVID-19, Health status, Workload, Lifestyle, Questionnaire survey

## Abstract

**Background:**

This study aimed to examine the change in health status of the general public after starting the COVID-19 pandemic and its association with changes in workload and lifestyle.

**Methods:**

A web-based survey was conducted in November 2020, about 9 months after starting the COVID-19 pandemic in Japan, among 8000 Japanese men and women aged 25–64 years. Participants asked for the changes after starting the COVID-19 pandemic in health status, workload, daily life, and health behavior. Ordinal logistic regression was performed to elucidate factors associated with deterioration in general health status.

**Results:**

A deterioration in general health status was reported by 17.0% of male and 19.4% of female. There has been a clear shift to sedentary life with decreasing moderate activity and increasing screen time. The multivariate analysis revealed that deteriorated work style, increased burden of housework, decreased moderate activity, increased digital media exposure, and increased body weight were significantly associated with deteriorating health status.

**Conclusion:**

Both men and women have experienced significant changes in workload and lifestyle since the COVID-19 pandemic started. People should be aware of the risks associated with their recent life changes and take self-care measures to prevent serious health consequences.

**Supplementary Information:**

The online version contains supplementary material available at 10.1186/s12199-021-00957-x.

## Introduction

The novel coronavirus, COVID-19, has raised serious concerns worldwide. COVID-19 spreads between people, mainly when an infected person is in close contact with another person [1]. The World Health Organization and many governments have called on people to implement personal protective measures, such as hand hygiene, cough etiquette, face masks, and social distancing [2]. Moreover, many countries imposed a full or partial lockdown to minimize person-to-person contact.

Since the COVID-19 pandemic started, people’s lives have been drastically changing. Daily schedule and lifestyle behavior were significantly different between before and during lockdown [3–6]. These changes are likely to have substantial impacts on people’s health [3]. Many epidemiological studies have been conducted to assess the impact of COVID-19 pandemic on the mental health in the general population. Systematic reviews and meta-analyses revealed that the COVID-19 pandemic was associated with highly significant levels of psychological distress that, in many cases, would meet the threshold for clinical relevance [7–9]. Since the COVID-19 pandemic is ongoing, it may bring further health problems both physically and mentally.

In Japan, COVID-19 was first detected in January 2020. The number of infected patients increased mainly in the Tokyo metropolitan area after a COVID-19 outbreak on a cruise ship in February 2020. The Japanese government declared a State of Emergency in response to the novel coronavirus disease on 16 April 2020. The first and second waves peaked in April and July, respectively, and another wave appears to be gaining momentum in November 2020. Although Japan has never imposed lockdown, the Japanese government has repeatedly and strongly urged the general public to implement personal protective measures. People are requested to avoid 3Cs (i.e., closed spaces, crowded places, and close-contact settings) and to refrain from face-to-face conversation without a face mask. The shift to remote working has been pressed forward in many companies to follow the stay-at-home request [10].

The drastic turn of people’s lives must have no small effect on people’s health. Because of the protracted and unpredictable nature of the COVID-19 pandemic, health professionals need to consider preventing health problems that can be caused by the lifestyle changes. However, few studies have focused on the changes in health status and lifestyle and their association in the Japanese population. In order to clarify this point, a web-based survey was conducted in November 2020, about 9 months after starting the COVID-19 pandemic in Japan. Information on changes in health status, workload, and lifestyle were collected, and the distribution of these changes was compared between men and women. Moreover, the association between deterioration in health status and changes in workload and lifestyle was assessed using multivariate analysis.

## Methods

A web-based survey was conducted in November 2020 among Japanese men and women aged 25–64 years, who were living in the metropolis of Tokyo and the three surrounding prefectures (Kanagawa, Saitama, and Chiba). In Japan, following the first wave in April and the second wave in July, a new wave of the COVID-19 pandemic appeared in November 2020, which was just before or after the survey.

The study protocol was approved by the ethics committees of the Jikei University School of Medicine (32-304 (10386)) and has been conducted in accordance with the Ethical Guidelines for Medical and Health Research Involving Human Subjects by the Japanese Government.

### Participants

Participants in the survey were recruited from an online research panel of a leading research company in Japan (Rakuten Insight Inc., Tokyo, Japan). Recruitment emails were sent to 61,129 randomly selected eligible registrants. Applicants for participation in the survey were accepted in the order of receipt until the number of participants reached the quotas (1000 people each) for gender and age groups (25–34, 35–44, 45–54, and 55–64 years old). A total of 9247 responses (15.1%) were obtained over 2 days of recruitment. All participants voluntarily agreed to participate in the survey after reading a description of the purpose and procedure of the survey. Consent to participate was implied by the completion and submission of the survey. Respondents received a reward in the form of “Rakuten Points” from the research company. The Rakuten Points earned can be used to shop on Rakuten Ichiba, to book accommodation through Rakuten Travel, and on other services across the Rakuten Group. After excluding those with incomplete or inconsistent answers to questions, a total of 8000 respondents were randomly selected from those with complete data and included in the study.

### Measures

On the web questionnaire pages, participants were asked about health status, attitudes and practices towards COVID-19, workload, daily life, health behavior, and communication with friends. They answered one question per page and could not go back to the previous page. The questions were prepared based on the questionnaires used in the SF-8 Health Survey (a question about general health status) [11], the Comprehensive Survey of Living Conditions (a question about diseases undergoing medical treatment) [12], the National Health and Nutrition Survey (questions about daily life and health behavior) [13], and previous epidemiological studies (questions about workload and daily life) [6, 14–16]. The components of the questionnaire relevant to this study are detailed below.

#### Health status

Participants were asked to rate their overall health on a six-point scale: excellent=5, very good=4, good=3, fair=2, bad=1, and very bad=0 [11]. For the changes in general health status after starting the COVID pandemic, participants chose one of five options (much improve, improve, no change, deteriorate, and much deteriorate), which were trichotomized into improve=1, no change=0, and deteriorate=−1 for analysis.

Participants were asked to check the disease(s) undergoing medical treatment on the disease list [12]. Those who had any disease(s) were further asked whether it was diagnosed before or after starting the COVID-19 pandemic.

#### Workload

Participants were asked whether they have changed jobs after starting the COVID-19 pandemic. Those who stayed in the same job were further asked whether their work style was improved or deteriorated after starting the COVID-19 pandemic.

Participants were asked whether the burden of housework increased or decreased after starting the COVID-19 pandemic. To come down to detail, the housework was resolved into homemaking, parenting, nursing, and caring, and participants evaluated the changes in these burdens, respectively.

#### Daily life

Participants were asked about the total time (hours) spent in moderate activity such as standing and walking, vigorous activity such as muscular labor, sedentary behavior such as sitting, and sleep in a weekday at the survey [13]. Additionally, TV viewing and digital media exposure by computers, tablets, and smartphones (only for private use) in the sedentary behavior were evaluated, because screen time tends to increase as a consequence of lockdown [6]. Participants were further asked whether this time increased or decreased after starting the COVID-19 pandemic, respectively.

#### Health behavior

Participants were asked about the following health behaviors at the survey: eating breakfast, eating between meals, eating after diner, drinking, and smoking [13]. Participants were further asked whether the frequency (or amount) of these behaviors increased or decreased, respectively, and whether their intention to change health behavior changed after starting the COVID-19 pandemic.

Participants were asked to report the change in body weight (kg) after starting the COVID-19 pandemic. Response options were ≤−5, −4≤ to ≤−2, −2< to <+2, +2≤ to ≤+4, and +5≤ kg.

### Statistical analysis

All statistical analyses were performed using the SAS ver. 9.4 (SAS Institute, Cary, NC, USA). The mean score of general health status were compared using two-way ANOVA with gender and age factors. The current status and changes after starting the COVID-19 pandemic in health status, workload, daily life, and health behavior were compared between men and women using chi-square test. The effect size for chi-square test was measured by Cramer’s V; a number between 0 and 1 indicates how strongly two categorical variables are associated. Ordinal logistic regression was performed to elucidate factors associated with deterioration in general health status. The objective variable was the changes in general health status (coded as improve=1, no change=0, and deteriorate=−1). The explanatory variables were the changes in occupation and work style (coded as improve=1, no change=0, and deteriorate=−1); burden of housework (coded as increase=1, no change=0, and decrease=−1); time spent in moderate activity, vigorous activity, sleep, TV viewing, and digital media exposure, respectively (coded as increase=1, no change=0, and decrease=−1); and body weight (coded as increase=1, no change=0, and decrease=−1). Odds ratios (OR) and 95% confidence intervals (CI) were calculated with adjustment for gender, age, occupation, marital status, income, and general health status at the survey. Significant levels were set at *p*<0.05.

## Results

Table 1 shows the characteristics of the study participants. The majority of participants had a university degree (male 69.0%, female 42.9%), were in full-time employment (male 78.9%. female 41.7%), were married (male 62.2%, female 63.1%), and had an adequate income (male 59.8%, female 52.1%). The proportions of university graduates to the study participants by gender and age group were considerably higher than those of the Japanese general population: According to the 2010 Japan Census, university graduates accounted for 31.9% and 23.8% in the 25–34 age group, 29.4% and 14.7% in the 35–44 age group, 32.9% and 12.4% in the 45–54, age group, and 24.2% and 7.0% in the 55–64 age group, respectively of the male and female population [17]. Meanwhile, the proportions of working people and married people to the study participants by gender and age group were roughly equal to those of the Japanese general population [17, 18].

**Table 1:**
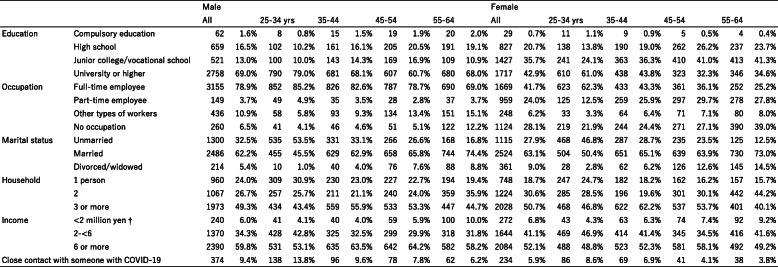
Characteristics of the study participants

^†^1 million yen was about 9600 US dollars at the time of the survey

Table 2 shows the change in workload after starting the COVID-19 pandemic. Those who have changed or lost their jobs were more frequently observed in female (15.6%) than in male (10.3%). The percentage of those whose work style deteriorated exceeded that of those whose work style improved in both genders. Those whose burden of housework increased were more frequently observed in female (36.7%) than in male (28.8%). The gender difference in burden of housework was especially prominent in parenting (Cramer’ V >0.1). Similar results were obtained from the analysis by age group (Supplementary table 1).

**Table 2:**
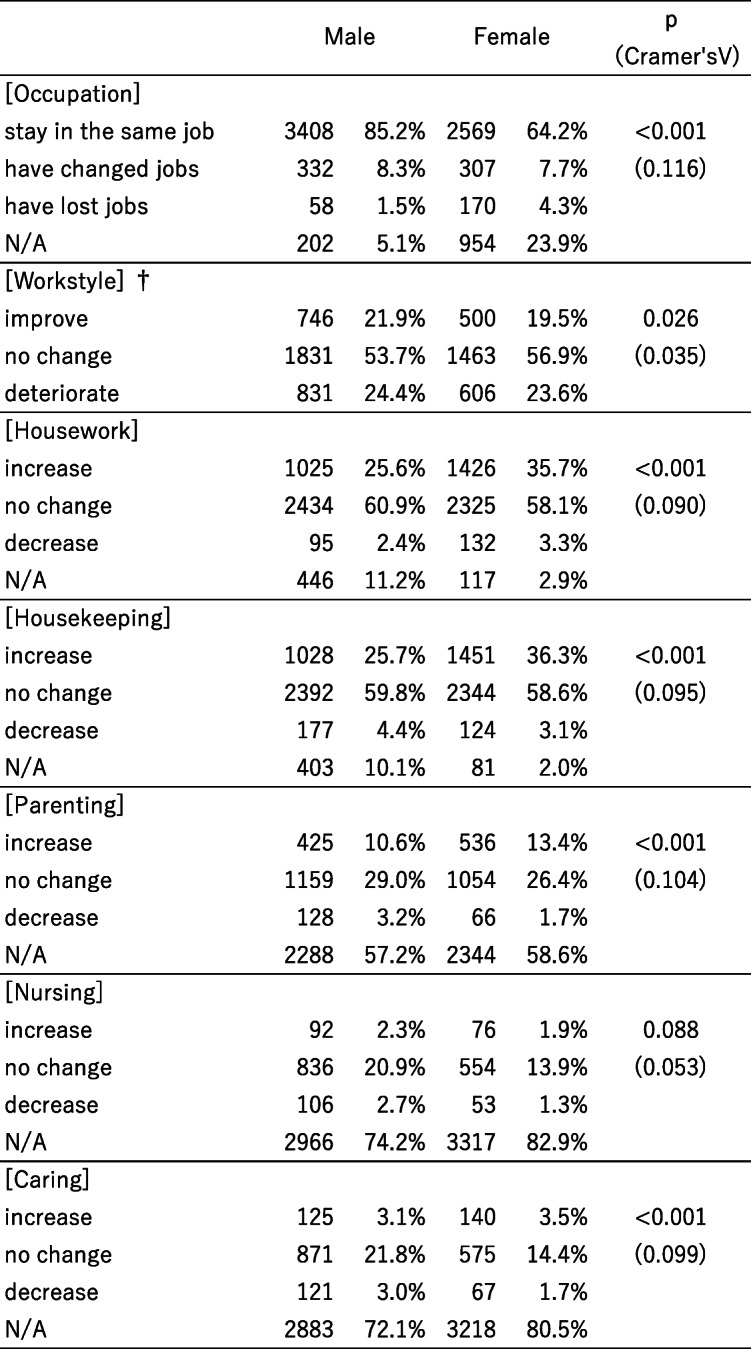
Change in workload after starting the COVID-19 pandemic

^†^Include only those who stay in the same job

Table 3 shows the change in daily life after starting the COVID-19 pandemic. One in every three participants reported that their time spent in sedentary behavior increased and that their time spent in moderate activity decreased in both genders. The majority of participants rarely or never engaged in vigorous activity and reported no change in the time spent in vigorous activity. In accordance with increasing time spent in sedentary behavior, those whose digital exposure increased accounted for more than 30% of participants in both genders. Those whose TV viewing increased were more frequently observed in female (27.3%) than in male (19.5%). The percentage of those whose sleep time increased exceeded that of those whose sleep time decreased in both genders. Similar results were obtained from the analysis by age group, excluding for the sleep time in the 55–64 age group (Supplementary table 2).

**Table 3:**
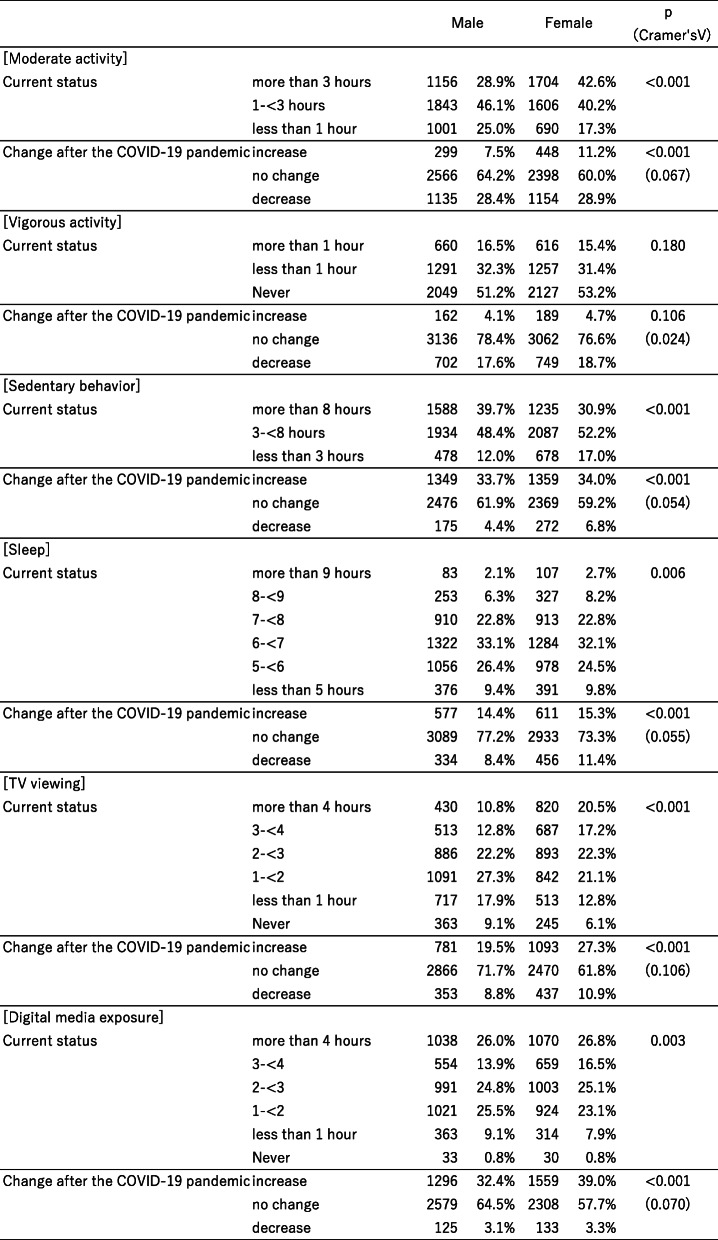
Daily life—current status and change after starting the COVID-19 pandemic

Table 4 shows the change in health status after starting the COVID-19 pandemic. One in every three participants reported that their overall health status was not good, having a score of less than 3. The percentage of those whose general health status deteriorated exceeded that of those whose general health status improved in both genders. Those who had any newly diagnosed diseases accounted for about 14% of participants in both genders. The most frequently reported disease in male was back pain, followed by dental diseases, hypertension, and stiff shoulder. The most frequently reported disease in female was gynecological diseases, followed by back pain, stiff shoulder, and dental diseases. The analysis by age group showed that the percentage of those whose general health status has improved was higher in younger age groups and that the percentage of those who had any newly diagnosed diseases was not different across age groups (Supplementary table 3).

**Table 4:**
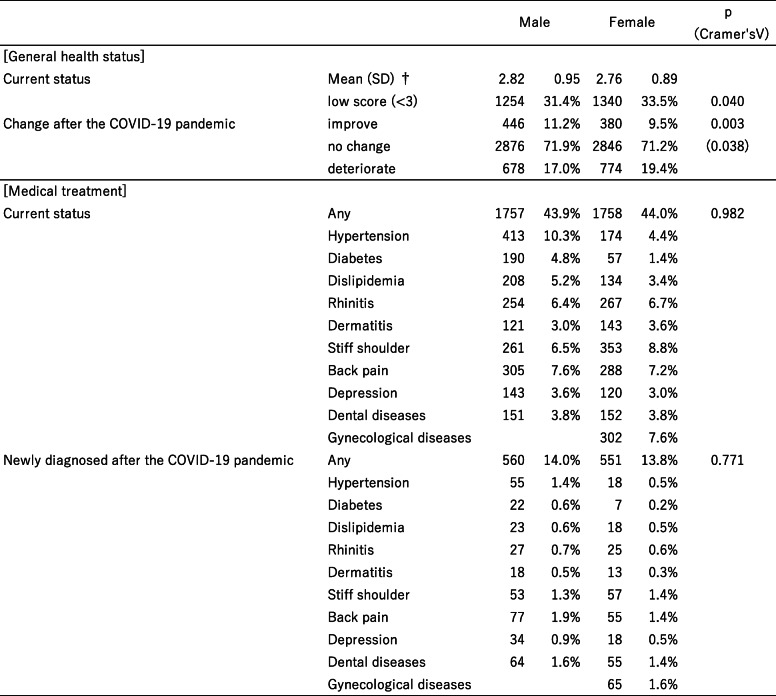
Health status—current status and change after starting the COVID-19 pandemic

The top10 most prevalent diseases undergoing medical treatment in the study participants were listed in the table

^†^Two-way ANOVA: gender *p*=0.004, age *p*=0.013, gender*age *p*=0.587

Table 5 shows the change in body weight and behavioral intention after starting the COVID-19 pandemic. One in every four participants reported their weight increased by 2kg or more, while one in every eight participants reported that their weight decreased by 2kg or more, in both genders. Those who developed an intention to change health behavior accounted for more than 40% of participants, and this percentage was higher in female than in male. Similar results were obtained from the analysis by age group (Supplementary table 4).

**Table 5:**
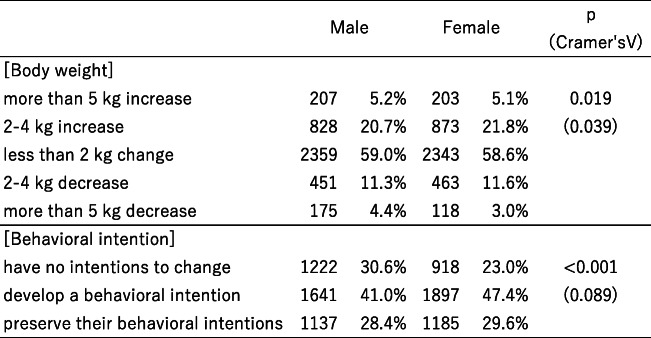
Change in body weight and behavioral intention after starting the COVID-19 pandemic

Table 6 shows the change in health behavior after starting the COVID-19 pandemic. The majority of participants reported no change in eating, drinking, and smoking habits in both genders. On the other hand, those who decreased or quitted drinking and smoking accounted for about 20% and 15% of participants, respectively in both genders. Similar results were obtained from the analysis by age group (Supplementary table 5).

**Table 6:**
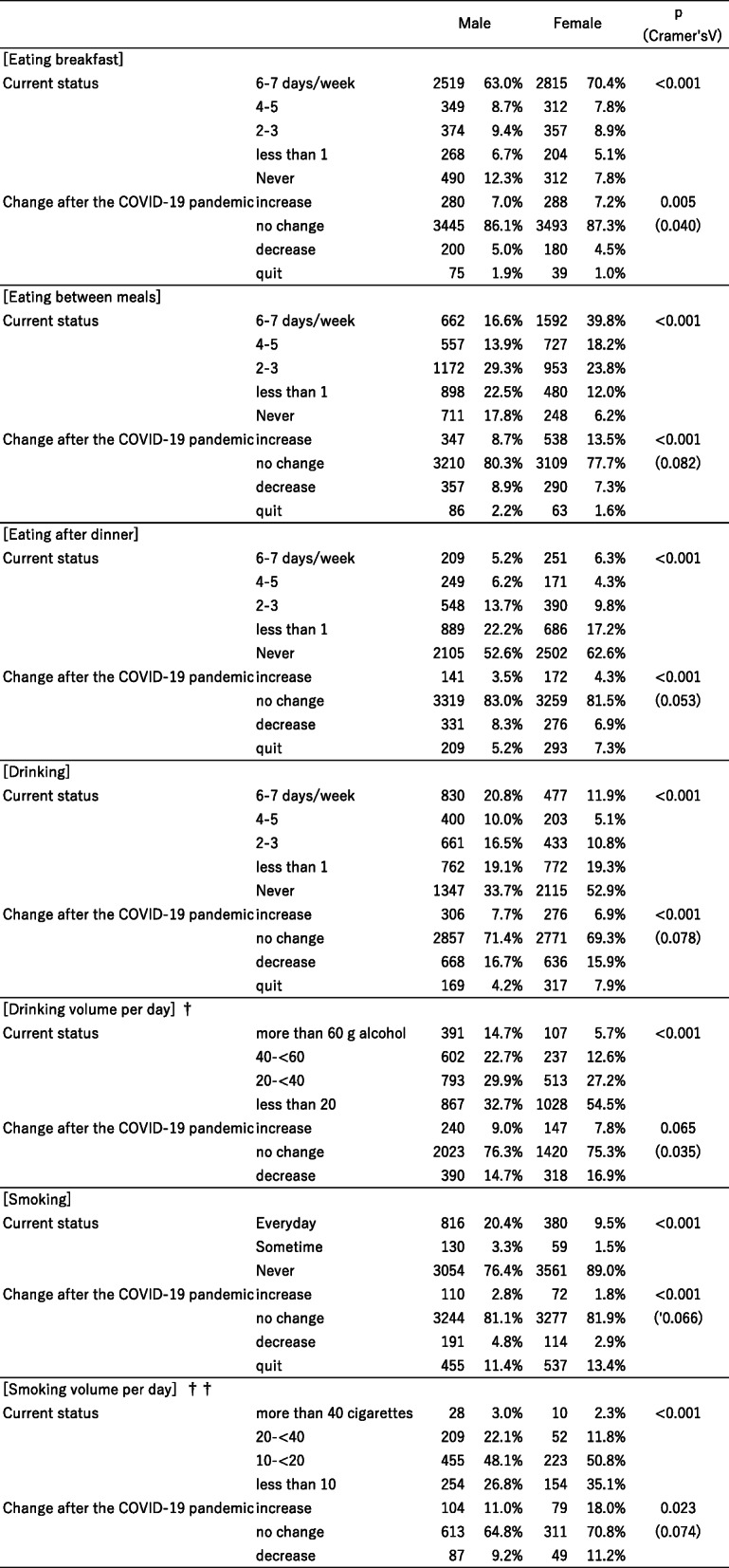
Health behavior—current status and change after starting the COVID-19 pandemic

^†^Exclude those who never drink

^††^Exclude those who never smoke

Table 7 shows the result of multivariate analysis which assessed the association of deterioration in general health status with changes in workload and lifestyle. The ORs significantly greater than 1 were found in deteriorated work style, increased burden of housework, increased digital media exposure, and increased body weight, while the ORs significantly smaller than 1 were found in job changing, increased moderate activity, and increased sleep time. When analyzed separately for male and female, the association of deterioration in general health status with increased burden of housework was significant in female but not in male.

**Table 7:**
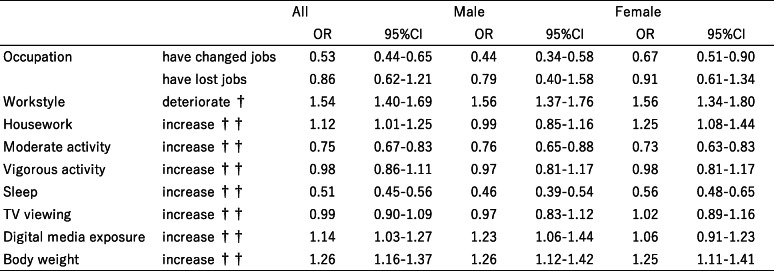
Factors associated with deterioration in general health status

Ordinal logistic regression was performed to calculate odds ratios (OR) and 95% confidence intervals (CI) with adjustment for gender, age, occupation, marital status, income, and general health status at the survey. Changes in general health status (i.e., objective variable) was coded as improve=1, no change=0, and deteriorate=−1

^†^Coded as deteriorate=1, no change=0, and improve=−1

^††^Coded as increase=1, no change=0, and decrease=−1

## Discussion

This study examined the change in health status and its association with changes in workload and lifestyle among 8000 Japanese men and women aged 25–64 years. As of November 2020, about 9 months after starting the COVID-19 pandemic in Japan, one in every three participants reported that their overall health status was not good. The percentage of those whose general health status deteriorated since the COVID-19 pandemic started (male 17.4%, female 19.4%) exceeded that of those whose general health status improved (male 11.2%, female 9.5%). The multivariate analysis revealed that the deterioration in general health status was significantly associated with changes in workload and lifestyle. Since the COVID-19 pandemic is ongoing, even subclinical changes in health status should not be ignored to prevent more serious consequences.

Among the workload factors, deteriorated work style and increased burden of housework were significantly associated with the deterioration of general health status. The majority of participants had not changed or lost their jobs. Among them, 24.4% of male and 23.6% of female reported that their work style deteriorated after starting the COVID-19 pandemic, which exceeded the percentages of those whose work style improved (male 21.9%, female 19.4%). Although the shift to remote working has been pressed forward in many companies, a large part of employees cannot have freedom to choose remote working [10]. Even though they received benefits from remote working, about 40% of employees expressed negative attitudes toward continuing this work style [10]. The work style changes due to the COVID-19 pandemic seem to have the potential to cause harm independently of increasing burden of housework. Further studies are needed to identify what kinds of work style changes contribute to deteriorating health status.

Among the lifestyle factors, decreased moderate activity and increased digital media exposure were significantly associated with the deterioration of general health status. This association was found independently of increasing body weight. The time spent in moderate activity was inversely related to the time spent in sedentary behavior; those whose moderate activity decreased and those whose sedentary behavior increased accounted for 28.4% and 33.7%, respectively of male and 28.9% and 34.0%, respectively of female. Therefore, it can be said that the shift to sedentary life during the COVID-19 pandemic was associated with deteriorating health status. It is well-known that physical inactivity increases the risk of many adverse health conditions and is also associated with poor mental wellbeing [19, 20]. Some epidemiological studies demonstrated a dose-response relationship between media exposure to information about COVID-19 and psychological symptoms in the general population [21, 22]. Those whose digital media exposure increased may have been affected by the media-fueled distress as well as the detrimental effects of physical inactivity. On the other hand, increased sleep time was significantly inversely associated with the deterioration of general health status. People with sleep deprivation account for about 70% of the Japanese population even in peacetime [13]. Since people have more time at home in response to the stay-at-home request from the government, making time for sleep has become easier. Getting enough sleep seems to have a protective effect against deteriorating health status due to the COVID-19 pandemic.

Although statistically significant differences between men and women were found in almost all change items, most of them were negligibly small (i.e., Cramer’ V <0.1), including the change in general health status (Cramer’ V 0.038). The changes in occupation (Cramer’ V 0.116), burden of parenting (Cramer’ V 0.104), and TV viewing time (Cramer’ V 0.106) barely showed meaningful gender differences. Stereotyped perceptions of gender roles, which assumes that men work outside the home while women remain at home doing housekeeping and parenting, seem to still persist in Japan [23]. Such gender inequality may have caused the higher percentages of those who have changed or lost their jobs, those whose burden of parenting increased, and those whose TV viewing increased in female than in male. As a consequence, the association of deterioration in general health status with increased burden of housework was significant in female but not in male.

This study provides evidence for the changes in health status, workload, and lifestyle after starting the COVID-19 pandemic in Japanese men and women. On the contrary, it has a number of potential limitations. First, the study participants were selected from registrants of a research company. People who cannot access the website through computers or smartphones have no opportunity to participate in web-based surveys. Those who are suffering from economic hardships, who have no digital devices, and who are not used to the Internet may not have been included in the study. Consequently, the study participants were more likely to be highly educated and of high socioeconomic status than the general population. Some epidemiological studies suggested that people with low socioeconomic status may have been hit harder by the COVID-19 pandemic [24, 25]. The impacts of the COVID-19 pandemic on the general population may be greater than those shown in this study. Second, the web-based survey was self-administered, so that the accuracy of responses would depend on participants’ motivation to answer questions accurately. Although the questionnaire was prepared carefully to minimize information bias, it is almost impossible to eliminate recall bias especially for the questions about the changes after starting the COVID-19 pandemic. Third, because of the cross-sectional design, this study helps to generate causal hypotheses but cannot prove causality. Further studies using longitudinal datasets are needed to confirm the causal relationship. Fourth, this study included people who were living in the metropolis of Tokyo and the three surrounding prefectures. There have been marked differences between urban and rural areas in the incidence of COVID-19, and the metropolis of Tokyo continues to record the greatest number of test-positives among 47 prefectures in Japan. It is uncertain whether the findings of this study are completely applicable to the population living in rural areas.

## Conclusion

A web-based survey was conducted in November 2020, about 9 months after starting the COVID-19 pandemic in Japan. About one in every five Japanese adults (17.0% of male and 19.4% of female) reported that their general health status has deteriorated since the COVID-19 pandemic started. Both men and women have experienced significant changes in workload and lifestyle, and these changes were significantly associated with deteriorating health status. People should be aware of the risks associated with their recent life changes and take self-care measures to prevent serious health consequences.

## Supplementary Information

**Additional file 1.** Supplementary tables

## Data Availability

The datasets generated and analyzed during the current study are not publicly available because the Ethical Guidelines prohibit researchers from providing their research data to other third-party individuals.

## Abbreviations

COVID-19: Novel coronavirus
OR: Odds ratio
CI: Confidence interval
