# Supplementary material for: Changes in health status, workload, and lifestyle after starting the COVID-19 pandemic: a web-based survey of Japanese men and women
Source: Environ Health Prev Med. 2021 Mar 22;26:37. doi: 10.1186/s12199-021-00957-x (PMC7982907; doi:10.1186/s12199-021-00957-x)
Supplement: Supplementary file 1 — Additional file 1. Supplementary tables [file 12199_2021_957_MOESM1_ESM.doc]

Supplementary table 1 Change in workload after starting the COVID-19 pandemic

Supplementary table 2 Daily life - current status and change after starting the COVID-19 pandemic

(to be continued)

Supplementary table 3 Health status - current status and change after starting the COVID-19 pandemic

The top10 most prevalent diseases undergoing medical treatment in the study participants were listed in the table.

† two-way ANOVA: gender p=0.004, age p=0.013, gender*age p=0.587

Supplementary table 5 Change in body weight and behavioral intention after starting the COVID-19 pandemic

Supplementary table 5 Health behavior - current status and change after starting the COVID-19 pandemic

(to be continued)

† exclude those who never drink.

†† exclude those who never smoke.
